# A short discourse on vascular tissue engineering

**DOI:** 10.1038/s41536-017-0011-6

**Published:** 2017-03-27

**Authors:** William G. Chang, Laura E. Niklason

**Affiliations:** Yale University, New Haven, CT USA

## Abstract

Vascular tissue engineering has significant potential to make a major impact on a wide array of clinical problems. Continued progress in understanding basic vascular biology will be invaluable in making further advancements. Past and current achievements in tissue engineering of microvasculature to perfuse organ specific constructs, small vessels for dialysis grafts, and modified synthetic and pediatric large caliber-vessel grafts will be discussed. An emphasis will be placed on clinical trial results with small and large-caliber vessel grafts. Challenges to achieving engineered constructs that satisfy the physiologic, immunologic, and manufacturing demands of engineered vasculature will be explored.

## Introduction

Vascular tissue engineering must translate the ever-expanding knowledge of vascular biology to develop new therapeutic options for a wide range of clinical disorders. Blood vessels serve as conduits to deliver oxygen and nutrients to, and waste products away from, tissues. They also must withstand a wide range of pressures and shear stresses, regulate blood flow and permeability, resist thromboses under basal conditions, and they also play critical roles in immunological responses.^1^ Blood vessels range in size and include microvessels (<1 mm), small vessels (1–6 mm), and large vessels (>6 mm in diameter).

In this review, we will highlight both historical and recent developments in tissue engineering of microvascular networks, and small and large-caliber vessels. It is not our intention to provide a comprehensive overview of this ever-growing field, but rather to introduce and stimulate interest in vascular tissue engineering. We will highlight progress toward clinical applications of tissue-engineered vasculature, and will discuss current challenges that must be overcome before more widespread use is possible.

Ideally, tissue engineered vasculature that is designed for therapy must be transplantable, or must stimulate the formation of new vasculature at the transplant site. Furthermore, engineered vessels for implantation must withstand physiological pressures without leakage or aneurysm formation, should not be thrombogenic, and should not elicit an immunological response from the patient. For clinical applications, the time that a patient must wait for a vascular therapy should be consistent with the clinical indication for use.

Concepts in vascular tissue engineering are broadly applicable to other areas of regenerative medicine. Rational use of synthetic and biological materials, and their combinations, will play a critical role in the progress of vascular tissue engineering. Functional anastomosis or inosculation to the patient, proper three-dimensional organization, and reproducible production at clinically relevant scales are important problems that must be addressed by vascular tissue engineers. Solutions to these problems will significantly advance tissue engineering and regenerative medicine overall.

### Microvascular tissue engineering

One of the fundamental hurdles that faces the rapidly evolving field of tissue engineering is supplying microvasculature to tissues that are too thick (>100–200 microns)^2^ to be maintained by diffusive nutrient transport alone. Producing a perfusable microvascular network is therefore critical to the treatment of ischemic disease and for the engineering of transplantable organs.^3^ Microvascular structures have been engineered by stimulation of angiogenesis in vivo, by implantation of endothelial cells, or by re-endothelialization of decellularized organs. In addition, microfabrication technologies may hold substantial promise for the future of in vivo vascular tissue engineering.

#### Stimulation of microvascular network formation in vivo

Stimulation of angiogenic responses in vivo has been examined clinically using a variety of cytokine-based,^4^ gene-based,^5^ and cell-based ^6, 7^ approaches. Several in-depth reviews of factors that stimulate pro-angiogenic responses have been published.^8–10^ In pre-clinical models, positive outcomes from stimulating angiogenesis have been shown with relatively simple interventions. One of the first examples of this was demonstrated when investigators gave a single intra-arterial bolus of vascular endothelial growth factor in a rabbit hind limb ischemia model to stimulate significant collateral vessel formation.^11^ Unfortunately, although animal studies have been promising, consistently beneficial pro-angiogenic drug therapies have not yet been developed for patients.^12^ For instance, plasmid based expression of FGF-1 showed clinical promise in patients with critical limb ischemia, when risk for amputation was assessed in a phase II trial in 125 patients.^13^ However, in a subsequent phase III trial that examined 525 patients, no significant improvements were noted when FGF-1 therapy was compared to placebo.^14^

Specific factors seen in elderly human patients that are not present in animal models may impair the efficacy of angiogenic therapies, such as underlying large vessel disease that could impair inflow, or deficiency of circulating or local vascular progenitor cells. Angiogenic interventions may need to be started early in the progression of critical limb ischemia in order to observe any benefits of therapy.^15^ The process of angiogenesis and vessel maturation and stabilization are complex processes that may not be fully recapitulated by the administration of one or two stimulating factors. Improving pro-angiogenic therapies by tightly controlling protein, gene, or cell delivery in a temporal manner is an area of active research that requires multidisciplinary approaches that incorporate biology, chemistry, and material sciences.

**Fig. 1:**
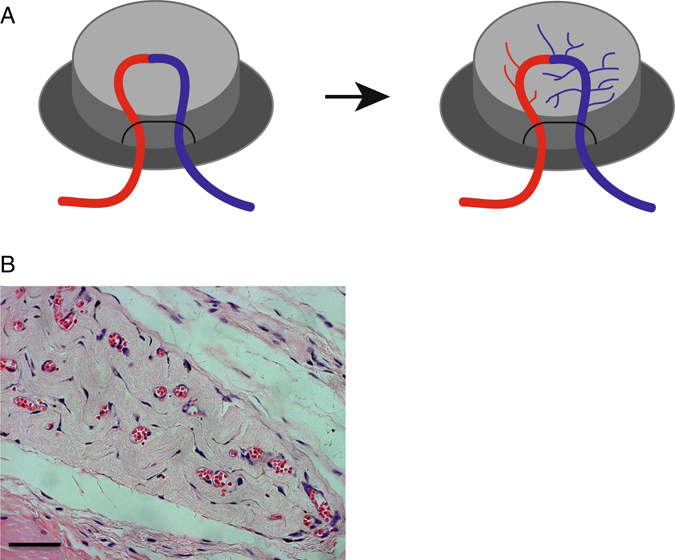
Approaches to in vivo microvasculature engineering. **a** Schematic of angiogenic arteriovenous loop. In vivo anastomosis between artery and vein is formed within a protected chamber that is fill with extracellular matrix. Angiogenic response is stimulated over time in vivo. **b** H&E of a section of collagen gel implant containing self-assembled, perfused microvessels after two weeks of subcutaneous implantation within an immunodeficient mouse. HUVEC and placental perictyes were freely suspended within the gels as decribed in [29] prior to implantations. Scale bar is 50 μm. Figure reproduced with permission from Oxford University Press

Another approach to stimulate microvessel formation that might support endogenous or transplanted tissues is to create an arteriovenous loop (AVL) with a very high blood flow rate. In a rat model, a femoral vein graft was interposed between the contralateral femoral artery and vein. The vein graft was then encased in a cylindrical chamber made from teflon or polycarbonate, that was filled with extracellular matrix and tissue-specific cells such as muscle,^16^ cardiac,^17^ or bone^18^ cells. Such AVL grafts create a significant local angiogenic response, with microvascular networks forming within the chamber that remained intact when assessed at 112 days.^19^ Protected AVLs have also been generated in large animals (Fig. 1a). In an ovine model, the saphenous vein and artery were directly anastomosed and enclosed within a Teflon chamber that was loaded with fibrin. AVLs were imaged at 1, 3, and 6 weeks with computed tomographic angiography (CTA) and magnetic resonance angiography (MRA). CTA demonstrated AVL patency in 5 out of 6 sheep, and 3D reconstructions of CTA scans demonstrated vascular sprouting predominantly from the venous side. This was supported by histological analysis of an explanted AVL at 6 weeks that demonstrated dense vascularization at the venous arm. Functional perfusion of the tissue chamber was further confirmed by MRA.^20^ This type of approach could be a clinically important one, but would be highly dependent upon type of tissue to be vascularized. Further pre-clinical work needs to be done.

#### Implantable engineered microvasculature

Vascular self-assembly also occurs with freely incorporated endothelial cells in an implanted matrix or scaffold. Human umbilical vein endothelial cells (HUVECs) undergo apoptosis when freely suspended in 3D collagen type I matrices,^21^ but survive when transduced with the anti-apoptotic protein Bcl-2. Robust microvascular networks supported by mural cells developed when hydrogels containing Bcl-2-transduced HUVECs were implanted subcutaneously into immunodeficient mice.^22^ Although this has not been observed experimentally, there is a theoretical concern that apoptosis-resistant cells may harbor some malignant potential if they were to be used clinically, and so extensive safety testing of such constructs would be necessary.

The process by which self-assembled endothelial cells inosculate with the host vasculature was examined by co-culturing HUVECs with mouse mesenchymal precursor (10T1/2) cells in collagen/fibronectin gels that were implanted in a cranial window preparation of immunodeficient mice. Implanted HUVECs self-assembled into microvessels that became perfused when they wrapped themselves around and then “tapped into” host vasculature at the edge of the implant.^23^ Microvessels that inosculated with the host in this manner were stable and functional for 1 year in vivo,^24^ thus demonstrating the stability of these engineered microvessels over time.

The cellular composition of engineered microvascular networks may be modified using different vascular cell sources. Endothelial cells can be derived from induced pluripotent stem cells,^25^ or may be supplemented with other supporting cells such as human bone marrow-derived mesenchymal stem cells,^26^ human smooth muscle cells,^27^ and human pericytes^28, 29^ (Fig. 1b). To generate an “immunoevasive endothelium”, microvascular networks were generated from endothelial cells that were genetically modified by clustered regularly interspaced short palindromic repeats (CRISPR)/CRISPR-associated protein 9 nuclease (Cas9). The class II major histocompatibility complex (MHC) transactivator (CIITA) was ablated^30^ in endothelial colony forming cells (ECFCs), which are endothelial progenitor cells. ECFCs have the proliferation potential necessary to clonally expand to very large cell numbers following the genetic modification, whereas typical HUVEC primary cultures do not have this clonal expansion capability. ECFCs were transduced with lentiviral vectors containing CRISPR/Cas9 elements to ablate CIITA, thereby rendering mutated cells unable to express MHC class II molecules. Engineered microvascular networks containing the modified ECFCs were protected from acute rejection when the immunodeficient murine hosts were challenged with human peripheral blood mononuclear cells that were allogeneic to the modified ECFCs.^31^ Therefore, “immunoevasive” microvascular tissue engineering has the potential to overcome a critical barrier to the vascularization of tissue engineered constructs—that of immune destruction of non-autologous microvasculature.

#### Combining microvasculature with engineered tissues

It is clear that perfusion of engineered tissues in vivo is necessary for the maintenance of any tissue of substantial thickness. Recent estimates for sprouting angiogenesis indicate a mean rate of sprout growth of around 5 μm/h in collagen in vitro.^32, 33^ Unless microvascular perfusion of implanted tissues can be augmented by approaches such as those described above, the bulk of an implanted tissue more than 1 mm in thickness will necrose before host-derived microvessels can perfuse the tissue.

Several studies indicate that in some cases, microvasculature can be combined with tissue specific engineered constructs to create viable implants. Reconstructed skin equivalents containing layers of fibroblasts, endothelial cells, and keratinocytes were generated to vascularize human skin-like grafts in immunodeficient mice.^34, 35^ These skin constructs inosculated with the host vasculature within 4 days. Constructs were cultured in vitro for at least 10 days prior to implant to allow for the pre-assembly of microvascular-like networks. Similarly, vascularized skeletal muscle-like constructs were generated by co-culture of endothelial cells, mouse myoblasts, and mouse embryonic fibroblasts within a porous biodegradable poly-(L-lactic acid) and polylactic-glycolic acid scaffold (1:1). These constructs were implanted subcutaneously in immunodeficient mice, intramuscularly in the quadriceps of nude rats, or as replacements of the anterior abdominal muscles of nude mice.^36^ Within these vascularized scaffolds, myocytes differentiated into multinucleated, elongated myotubes after 2 weeks in vivo. In some cases, the myotubes seemed to align with the host muscle fibers. Further functional characterization of the muscle was not performed in this study, but vascularization and implant survival were clearly improved by the addition of endothelial cells within the constructs prior to implant. These two approaches show promise for the development of clinically useful vascularized skin and muscle constructs for the treatment of superficial and deep wounds, traumatic muscle volume loss, or muscular dystrophy.

Engineered microvasculature can also be used perfuse stem cell derived human organoids. Human iPSC-derived hepatic cells that were co-cultured with mesenchymal stem cells and HUVECs resulted in liver buds that could be perfused by the host vasculature when implanted into an immunodeficient mouse. The resulting liver tissue produced human albumin and could metabolize drugs in vivo,^37, 38^ but the cells did not form cholangiocytes (biliary duct epithelium), which are necessary to clear toxic byproducts of hepatic metabolism. Vascular density was significantly enhanced by the addition of the iPSC-derived hepatic cells when compared to implants containing only endothelial cells and mesenchymal stem cells. This suggests that the liver cells themselves had a stimulatory effect on microvessel formation. Despite this progress, at present the vascularization of parenchymal cells is not enough to recapitulate the full function of complex organs such as the liver.

Collectively, these investigations demonstrate that engineered microvasculature can be successfully utilized to perfuse collections of cells that are grafted in vivo. Further work needs to be done to expand the mass of cells that can be supported by engineered microvasculature, and thereby enhance the size and functionality of implantable tissues. One clear limitation with the AVL model and gel-based endothelial microvascular self-assembly is that there is limited control over the 3D orientations of the vessels formed. This control may be achieved by relying upon the native organ scaffold or by advances in microfabrication technologies.

#### Decellularization and recellularization

To mimic the natural vascular organization of a specific organ, decellularization techniques have been employed. Typically using mild detergent solutions, DNAase applications and extensive washing, decellularization regimens have been developed that maintain the extracellular matrix scaffold of the organ, including microvascular basement membranes. Decellularization approaches for heart,^39^ lung,^40, 41^ kidney,^42^ and liver^43^ have all been described. Decellularization that does not cause disruption of vascular channel integrity is paramount for these approaches to generate matrices that are suitable for cellular repopulation. In addition, efficient endothelial repopulation of acellular constructs—using autologous or allogeneic endothelium—is necessary to prevent microvascular coagulation stimulated by exposed collagenous matrix. Furthermore, comprehensive endothelial repopulation, contributing barrier function to the niche of parenchymal cells within the organ, is a critical and still non-realized aspect of whole organ recellularization. At present, the AVL strategy, gel-based endothelial self-assembly, and decellularization-recellularization strategies appear to be the most promising routes to achieving robust microvascular perfusion of tissue engineered organs, but new microfabrication approaches are quickly developing as well.

#### Micropatterning approaches to microvessel creation

To produce highly controlled and reproducible patterns of microvasculature, microfabrication techniques are also being developed. These approaches are not yet ready for clinical application, but nevertheless are important developments in vascular tissue engineering that will likely shape future directions.

Recent developments in microfluidics and 3D bioprinting have generated technologies that are useful as testbeds for investigating both vascular biology and tissue engineering applications.^44^ Three commonly utilized microfluidic approaches include utilizing sacrificial templates, micropatterning using soft lithographic techniques, and in vitro endothelial self-assembly. One major challenge will be how to translate these new technologies to clinically impactful vascularization of tissue engineered constructs.

**Fig. 2:**
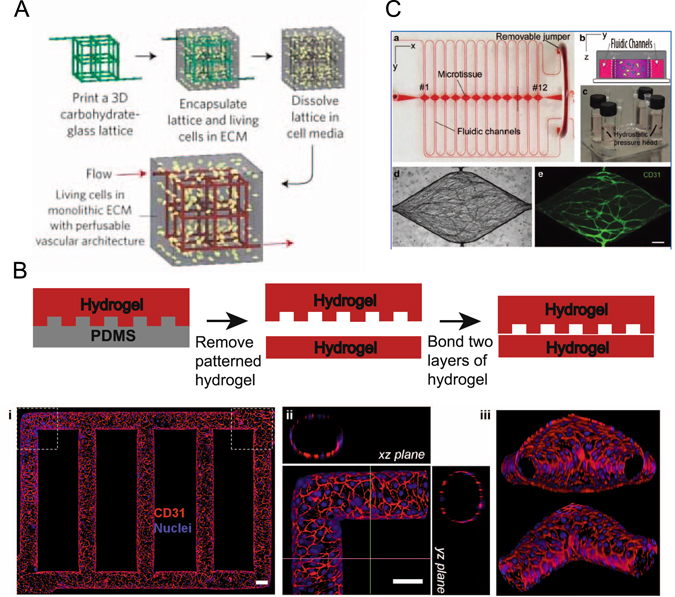
Microfluidic approaches to in vitro microvascular engineering. **a** Schematic of self-supporting 3D printed carbohydrate-glass lattice that is encapsulated. Once the lattice is dissolved, a perfusable network results. Living cells can be placed within the ectracellular matrix and within the perfusable channels. **b** (*Left*) Schematic protocol of microfluidic device construction by bonding micropatterned hydrogel and thin layer to form microvascular channel network. (*Right*) Top and cross-sectional views of confocal *Z*-stacks showing endothelial-lined microvascular channels. Red is CD31 and blue are nuclei. Scale bar is 100 μm. **c** (*Top*) Microfluidic platform made from PDMS that consists of a daisy chain of microtissue chambers connected by pores and loaded with extracellular matrix, endothelial cells, and fibroblasts. (*Bottom*) Brightfield and CD31 immunofluroescent staining demonstrating microvascular networks formed by endothelial self-assembly. Scale bar is 200 μm. Figures reproduced and modified from [46, 47, 48] with permissions from Nature publishing, PNAS, Tissue engineering part **c**. Scale bar is 50 μm

Sacrificial approaches include casting hydrogels around sacrificial struts that leave perfusable channels that can be seeded with endothelial cells when removed.^44, 45^ Dissolvable materials such as carbohydrate glass filaments can be 3D printed into lattices, and surrounded by extracellular matrix. After the glass filaments are dissolved, the 3D lattice could be perfused and loaded with cells^46^ (Fig. 2a). One significant advantage of this approach is that the size and scale of the 3D lattices can be easily scaled and controlled. Another approach for patterned vascular structures uses lithography to micropattern a hydrogel that forms a microvascular network when bonded to another flat layer of hydrogel (Fig. 2b).^47^ Others have developed approaches to harness the capacity for endothelial cells to self-assemble into microvascular networks, by applying interstitial flow to extracellular matrix-containing mixtures of endothelial cells and mural cells within microfluidic chambers^48, 49^ (Fig. 2c). Due to the small scales (typically volumes <1 mm^3^) and easy visualization of these platforms, the strength of these approaches thus far is to facilitate more efficient analyses of vascular biology, disease modeling, or drug testing. One question is whether microvessels that are formed in the presence of in vitro flow can enhance stabilization or functionality of microvessels to be implanted in vivo at later times. With continuing improvements in the spatial resolution of 3D bioprinting, which incorporates printed cells and biocompatible matrices, new options will become available for achieving tight control of vascular and tissue-specific structures.^50^ We suspect that it may be necessary to combine several techniques to achieve adequate perfusable microvasculature in large engineered constructs containing functional tissue specific parenchymal cells.

### Small vessel tissue engineering

For small caliber arteries (<5–6 mm in diameter), synthetic grafts have unacceptably high failure rates, with peripheral graft patency of <25% at 3 years as compared to >70% for autologous vein.^51^ Furthermore, there are essentially no non-autologous conduits that are useful in the coronary circulation, and synthetic arterio-venous grafts used for dialysis access have very high failure rates. There is clearly a significant need to develop engineered small diameter grafts to address a range of clinical scenarios.

One of the early attempts at engineering arterial replacements was to generate autogenous conduits made of fibrous tissue, by subcutaneously implanting a 5.1 mm diameter mandrel into the patient. The mandrel was covered by loosely knitted Dacron (polyethylene terephthalate synthetic fiber) that was surrounded by an outer tube with multiple spaces for cell infiltration. After 5–12 weeks of implantation, the fibrous tissue formation encasing the mandrel led to the formation of the “Sparks’ Mandrel” construct (named after its inventor Dr. Charles Sparks).^52^ The fibrous tubes were harvested from their subcutaneous sites and implanted as arterial bypass grafts, typically in the thigh, but several difficulties were noted. These grafts did not always mature efficiently in the subcutaneous space, and early and late thrombosis occurred frequently. In addition, aneurysms formed in approximately 20% of cases.^53^ Therefore, this type of technology was not effective enough for continued clinical use, and has largely been abandoned, though some efforts in this arena are ongoing.^54–56^

The first attempts to generate a small caliber-vessel in vitro were published in 1986. Smooth muscle cells in a collagen gel were cast around a central mandrel to mimic the medial vascular layer of a muscular artery. After 1 week, the collagen gel layer was further supported by a Dacron mesh sleeve. The outer layer was cast with fibroblasts and incubated for an additional two weeks to form an adventitial-like layer. Finally, the tubular structure was slipped off the mandrel and endothelial cells were seeded onto the lumen for 1 week. Although an endothelial layer was formed that could release prostacyclin to inhibit platelet aggregation, the burst strength achieved with these grafts was only ~300 mm Hg,^57^ which is too low to withstand arterial implantation. Although this type of engineered artery was never implanted successfully in vivo, this work was important conceptually for the field, since it served as a starting place for co-culturing endothelial cells and supporting mural cells to engineer artery-like structures.

Other investigators have used fibroblasts cultured in fibrin hydrogels to generate small diameter vascular grafts. In this system, the hydrogels containing fibroblasts were cast into a tubular mold containing a central glass mandrel. Cultured fibroblasts were used to generate extracellular matrix, predominantly collagen. Grafts were cultured for 7–9 weeks in a pulsatile flow-stretch bioreactor, to induce circumferential alignment of the cells and the matrix.^58^ In subsequent reports, engineered vessels constructed in a similar manner were decellularized using 1% sodium dodecyl sulfate and implanted as interpositional grafts in sheep femoral arteries. These engineered arteries achieved complete endothelialization, and showed evidence of elastin deposition at 24 weeks.^59^ The results from this approach are encouraging, and may result in clinical translation at some point in the future.

Additionally, a few approaches for small and large vessel engineering have progressed to clinical trials. Human, completely autologous engineered grafts have been generated from skin fibroblast cell sheets, and have been implanted into patients as arteriovenous grafts for hemodialysis access. In a multicenter cohort study in Argentina and Poland, ten end-stage renal disease (ESRD) patients underwent skin biopsies to procure autologous fibroblasts, which were used to generate cell sheets grown in vitro (Cytograft, Inc.). Sheets were wrapped around a 4.7 mm mandrel, and the layers of the sheets allowed to fuse in a bioreactor for 10 weeks. Endothelial cells isolated from the patient’s own superficial veins were then seeded into the lumens of the grafts, to produce completely autologous conduits. Of note, production times were between 6–10 months to generate these autologous engineered vessels. In the study of 10 patients, one patient withdrew prior to implantation, and of the remaining 9 patients, three grafts failed during the initial 3 months because of a graft dilatation, aneurysm, or thrombosis. One patient died of cardiac failure secondary to pneumonia, while the five remaining patients were able to continue dialysis for >6 months.^60^ In a more recent update, of 13 total grafts placed, 4 failed within the first 90 days, most commonly due to mechanical defects.^61^ Failure modes of these grafts appear overall similar to those reported for the Sparks’ mandrel grafts, and it is unclear if further clinical trials will be undertaken with these constructs.

Studies in our group, initiated in the 1990’s, utilized bioreactors for culturing engineered arteries that were capable of introducing pulsatile flow and radial strain. Vascular smooth muscle cells were seeded onto a biodegradable polyglycolic acid (PGA) scaffold, and grown in culture medium that contained growth factors and biochemical supplements to support collagenous extracellular matrix synthesis. The cyclic mechanical strain delivered by the bioreactors during vessel culture was designed to mimic the mechanical environment of native arteries. Seeded scaffolds underwent pulsatile radial stretch for 8 weeks, after which endothelial cells were seeded onto the graft lumens and were cultured for 3 more days. Engineered arteries achieved burst pressures of >2000 mm Hg. Autologous engineered grafts were implanted successfully into the right saphenous artery in a miniature swine model, with patency for up to 24 days.^62^ This report by our group, published in 1999, was the first demonstration of the feasibility of culturing autologous, implantable arteries that were functional in a large animal model.

**Fig. 3:**
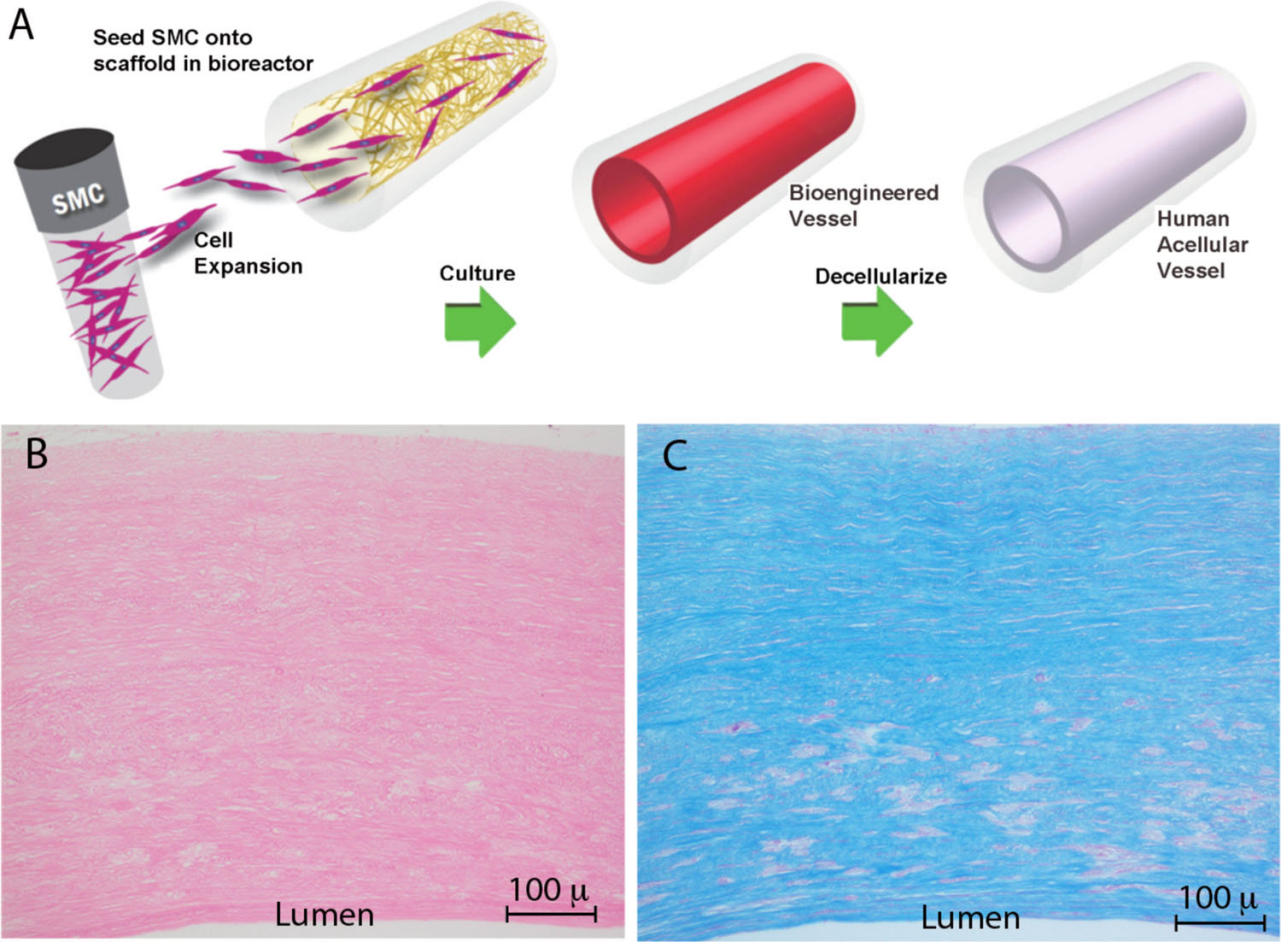
Engineered acelluar small-vessel grafts. **a** Schematic demonstrating steps in the construction of acellular vascular grafts. Cadaver-derived SMCs are seeded onto tubular PGA scaffold, which are then cultured in bioreactors. PGA degrades leaving, collagenous tissues which is then decellularized for “off the shelf” vascular graft. **b** Hematoxylin and eosin and **c** Masson’s trichrome collagen staining of decellularized graft. Scale bars are 100 μm

In an extension of this approach, to generate engineered arteries that could be more rapidly used for human implantation, human organ donor-derived SMCs were seeded onto PGA scaffolds and then cultured in bioreactors. After 8 weeks of culture, the cellular and collagenous tissue that formed was then decellularized, in order to remove allogeneic cellular antigens. Such acellular grafts could then be immediately available, “off the shelf”, for vascular grafting applications that bear a relatively low risk of thrombosis (Fig. 3).^51^ Produced by Humacyte Inc., these human acellular vascular grafts have been implanted into 60 ESRD patients for two single-arm phase II trials at six centers in the US (20 patients) and Poland (40 patients) with a mean follow-up of 16 months.^63^ At 12 months, 28% of the 60 study patients had primary patency (functional access patency without intervention) and 89% had secondary patency (functional patency with or without intervention). At 18 months, primary patency was 18% and secondary patency was 81%. By means of comparison, historically, synthetic ePTFE grafts have approximately 33% primary and 55% secondary patencies at 18 months.^64^ In contrast to the Sparks’ mandrel grafts, no aneurysms were reported with these acellular grafts, and no evidence of a significant immunological response or rejection was noted. The acellular engineered arteries appear to repopulate with cells from the recipients over time. One engineered vessel that was partially biopsied at 16 weeks showed host cell re-population with smooth muscle cells or myofibroblasts, and with endothelial cells on the lumen. This host cell repopulation appeared to progress over time, with another specimen obtained at 55 weeks showing more extensive host cell repopulation, and little inflammatory response. A phase III clinical trial is ongoing to directly compare these acellular engineered grafts with expanded polyterafluoroethylene (ePTFE) dialysis grafts in an arteriovenous setting. If significant clinical improvements in functional patency and/or infection rates are noted when acellular engineered graft are compared to ePTFE grafts, this would represent a critical step toward making engineered grafts a valuable new option for dialysis patients.

Tissue engineering of small-caliber vascular grafts remains a significant challenge, yet clinical trials with products from both Cytograft and Humacyte, Inc. suggest that routine clinical application of engineered vessel technologies may be close to becoming a reality, especially for the ESRD patient population. More work is required to determine whether any of these approaches can be used to provide new options to those patients needing vessels for coronary artery bypass procedures and other small-caliber, low blood flow scenarios such as distal extremity arterial bypass. Successful translation of engineered graft technology to dialysis grafts would be an important proof-of-principle for future clinical applications such as coronary bypass grafting.

### Modified synthetic and pediatric large vessels tissue engineering

For large caliber vessels (>6 mm), synthetic materials like Dacron^®^ (polyethylene terephthalate) and Goretex^®^ (polytetrafluoroethylene) have been relatively successful for aortic, iliac, and common femoral artery repairs.^65^ However, biological modifications of synthetic grafts are being further explored to enhance efficacy. For instance, the FUSION BIOLINE heparin-coated vascular graft (produced by MAQUET Cardiovascular, LLC) was compared to ePTFE grafts for above and below knee femoropopliteal bypass that were 6 and 8 mm in diameter in a multicenter, randomized, and controlled trial with 203 patients. Modified grafts consisted of an inner layer of ePTFE with a bioactive surface coating containing heparin sodium coupled to recombinant human albumin. The outer layer contained Dacron that was fused to the inner modified ePTFE with polycarbonate-urethane adhesive. Primary patency at 6 months was 86.4% for the heparin modified graft and 70% in the standard ePFTE group (*p* = 0.006). However, the difference in primary patency was less apparent at 12 months of follow-up (76.5% vs. 67% with a *p* = 0.05).^66^ Further follow-up will be needed to assess whether improved patency will be sustained past 1 year, but this example illustrates how both material and biological modifications to a graft may yield improved clinical outcomes.

Another approach that combines synthetic and biological modifications to large-caliber vessel engineering is currently being explored in pediatric patients with congenital heart disease. This scenario is clinically important because synthetic graft materials lack the ability to grow with their pediatric recipients. Therefore, reconstructive surgery of congenital vascular anomalies is sometimes delayed until the patient has grown large enough for an adult-sized graft. Alternatively, an oversized graft is implanted in the pediatric recipient, or repeated operations are required as the child grows. Hence, an engineered blood vessel that could grow with the pediatric patient would be ideal for repair of congenital cardiovascular defects, and could be transformative for the care of these rare patients.

Studies in large animals support the growth potential of engineered vascular grafts. In a juvenile lamb model, investigators seed autologous bone marrow derived mononuclear cells onto degradable non-woven PGA mesh tubes coated with caprolactone/L-lactide co-polymer that then were implanted into the inferior vena cava. Seven animals were examined using serial, bimonthly MRA over a 6-month period. Graft volumes increased ~127% over 6 months, which was roughly proportional to the growth of the native pulmonary artery. Histological analysis of the explanted conduits demonstrated endothelial lining supported by several layers of smooth muscle cells arranged concentrically,^67^ supporting the extensive remodeling and growth potential of these implants.

An engineered blood vessel with this growth potential was first reported in a human in 2001.^68^ A 4-year-old girl with a single right ventricle and pulmonary atresia, status post pulmonary artery angioplasty and a Fontan procedure at age 3, was found to have a total occlusion of the right intermediate pulmonary artery. A small segment of a peripheral vein was explanted to isolate and expand a mixed population of cells (mostly myofibroblasts and smooth muscle cells) over 8 weeks. A 1 cm diameter biodegradable tube that was made from caprolactone/lactide/PGA copolymer was seeded with the autologous cells derived from a peripheral vein and cultured for 1 week to generate an engineered graft. Follow-up angiography showed patency and after 7 months of follow-up, the patient was doing well without signs of graft occlusion or aneurysmal changes on chest radiography.

In subsequent clinical applications of this approach, the patients’ own bone marrow-derived cells were used, rather than mixed cells derived from peripheral veins, for scaffold repopulation. As a rationale for this change, the investigators cited a study that demonstrated that infilitration of bone marrow cells in a dog model of ePTFE vascular grafts yielded good endothelialization after implantation.^69^ In further efforts to streamline this approach, harvested bone marrow-derived cells were incubated on the grafts for only 2–4 h before implantation into the recipient. The removal of the cell expansion step reduced the time needed to make these grafts, as well as the risks for bacterial contamination.^70^ In a Japanese cohort of 25 patients with a median age of 5.5 years and a mean follow-up of 5.8 years, all grafts were patent by imaging at 30 days of follow-up, without evidence of stenosis, thrombosis, or aneurysmal dilation. At 1-year follow-up, one patient developed a thrombosis that was treated with warfarin for anticoagulation. At late-term follow-up, six patients of the 25 had asymptomatic graft narrowing, and four of those had successful angioplasty to treat these stenoses.^71^ As a follow-up to this report, there is a nonrandomized Phase I clinical trial in the US that is looking at the safety of these engineered vessels in pediatric patients that is currently ongoing.

In an effort to understand the remodeling and occasional stenosis seen in the pediatric patients, murine models have been utilized to dissect cell populations that affect the remodeling response. It was observed that the bone marrow cells that were initially seeded onto the polymer grafts were not, in fact, incorporated into the remodeled vessel in vivo over time. Rather, host cells appeared to be recruited to populate the synthetic scaffold. In particular, monocytes recruited by the secretion of MCP-1 by the seeded bone marrow derived cells likely played a major role in vessel remodeling.^72^ Whether these MCP-1 recruited cells are the primary effectors in graft stenosis is not yet clear, however.

The clinical results for arteriovenous access and for pediatric reconstructive surgery are encouraging, and demonstrate that vessel engineering is progressing from a laboratory-based academic endeavor to one that is impacting the lives of patients having a range of diseases. However, further confirmation of graft efficacy, safety, and long-term function are still required, and will be assessed in additional controlled studies.

## Conclusion

It is clear that continued investigations into basic vascular biology will yield greater insights into how engineered constructs—both microvascular implants and engineered conduits—can be improved further. Interactions between endothelial cells and mural supporting cells will need to be understood more completely in order to direct and control remodeling and host inosculation. The era of personalized medicine enabled by stem cell technology, and new approaches to genetically modify implanted cells, have the potential to generate engineered constructs that can avoid immunological rejection. Efforts to shorten manufacturing times and produce constructs that are “immunoevasive” will likely lower cost and increase the “off-the-shelf” availability for several types of vascular replacements. Finally, recently completed clinical trials, and other trials that are underway or planned for the near future, seem to show that we are entering a new era of therapy for vascular disease, that employs cells and engineered tissues in the treatment of human disease.
